# Physiological Changes in QRS Fragmentation in Athletes and Nonathletes without Cardiac Disease

**DOI:** 10.3390/jcm13102741

**Published:** 2024-05-07

**Authors:** Georgios A. Christou, Maria A. Christou, Konstantinos A. Christou, Dimitrios K. Christodoulou, Dimitrios N. Kiortsis

**Affiliations:** 1 Department of Radiology, Faculty of Medicine, University of Ioannina, 45332 Ioannina, Greece; 2 Atherothrombosis Research Centre, Faculty of Medicine, University of Ioannina, 45332 Ioannina, Greece

**Keywords:** QRS fragmentation, athletes, body mass index, inspiration

## Abstract

**Background/Objectives:** QRS fragmentation has not been linked with increased mortality in individuals without known cardiac disease. We aimed to investigate the physiological determinants of QRS fragmentation in individuals without cardiac disease. **Methods:** Study participants were 163 (54 athletes, 109 nonathletes) asymptomatic individuals with QRS fragmentation but without cardiac disease. QRS fragmentation was assessed in the supine position after deep inspiration or standing up and during exercise. The changes in QRS fragmentation were evaluated over a median follow-up period of 2.3 (0.8–4.9) years. **Results:** The most common lead with QRS fragmentation was III (63.0% in athletes, 61.5% in nonathletes), immediately followed by V1 (50.0%) and aVF (42.6%) in athletes and aVF (55.0%) in nonathletes. QRS fragmentation in V1 was more frequent in athletes compared to nonathletes (*p* < 0.001). Among athletes, the presence of QRS fragmentation in V1 could be independently predicted by increased RVOTproxi (right ventricular outflow tract proximal diameter indexed to body surface area) (*p* < 0.001). Among individuals with QRS fragmentation in V1, deep inspiration resulted in disappearance of QRS fragmentation more frequently in nonathletes compared to athletes (100% vs. 20%, *p* = 0.003). Deep inspiration resulted in disappearance of QRS fragmentation in aVF (*p* < 0.001). The presence of QRS fragmentation in II or aVF was associated with increased body mass index (BMI) (*p* = 0.003). Among athletes without QRS fragmentation in V1 at baseline, the appearance of QRS fragmentation in V1 at the end of follow-up was associated with greater training age (*p* = 0.034). Among individuals with QRS fragmentation in aVF at baseline, the disappearance of QRS fragmentation in aVF at the end of follow-up was associated with greater reduction in BMI (*p* = 0.008). **Conclusions:** The characteristic feature of QRS fragmentation in athletes was the presence of QRS fragmentation in V1, which was associated with RVOTproxi. The persistence of QRS fragmentation in V1 after deep inspiration could serve as a specific marker of exercise-training-related cardiac adaptation. The presence of QRS fragmentation in the leads of the frontal plane was influenced by BMI and respiration phase.

## 1. Introduction

Fragmented QRS is traditionally defined as an additional notch in the QRS complex in the absence of bundle branch block [1,2,3]. The electrophysiological mechanism underlying the pathogenesis of QRS fragmentation has been found to be the existence of complex and nonuniform pathways of myocardial excitation [4,5].

The presence of QRS fragmentation in individuals with structural heart disease has been reported to be associated with the existence of myocardial fibrosis and with adverse cardiac events [3,6]. However, QRS fragmentation has not been linked with increased mortality in individuals without known cardiac disease, suggesting the possible benign nature of QRS fragmentation in apparently healthy individuals [6,7]. In this respect, the identification of the physiological determinants of QRS fragmentation in ostensibly healthy individuals can aid in the correct interpretation of this electrocardiographic pattern and also shed light on which dynamic changes in QRS fragmentation could be normally expected or alternatively may indicate a sign of underlying cardiac pathology. These considerations are particularly important in athletes, since the possibility of chronic exercise-related electrocardiographic patterns should always be taken into account [8]. Even more, athletic activity poses an additional risk of sudden cardiac death in individuals with concealed cardiac disease, mandating a low threshold for extensive diagnostic evaluation in case of suspicious electrocardiographic patterns [9,10].

However, relatively few studies have explored the clinical significance of QRS fragmentation in athletes, since the overwhelming majority of the medical literature investigating QRS fragmentation has focused on populations with known cardiac disease [1,11,12]. A limitation of the studies having investigated QRS fragmentation in athletes so far was that they analyzed the prevalence of QRS fragmentation only in groups of contiguous electrocardiographic leads, essentially leaving unexplored the clinical significance of QRS fragmentation in each specific lead, with the only exception of V1, the QRS fragmentation of which was defined in a restrictive way as the presence of quadriphasic QRS complex without including cases with notched QRS in V1 [1,11,12].

The physiological alterations in the position of the heart after deep inspiration, change in posture from supine to standing, and change in body weight could be associated with physiological changes in QRS fragmentation [13,14,15]. Moreover, the change in sympathathovagal balance during exercise may influence intraventricular conduction and thus the presence of QRS fragmentation during exercise testing [16].

Therefore, the aim of the current study was to explore the distribution of QRS fragmentation in each specific electrocardiographic lead in athletes and nonathletes without cardiac disease and unravel the physiological determinants of QRS fragmentation. Furthermore, we aimed to investigate for the first time the alterations in QRS fragmentation following deep inspiration or standing up, which are known to modify the position of the heart, the dynamic changes in QRS fragmentation during exercise, and the physiological long-term variations in QRS fragmentation over time.

## 2. Materials and Methods

### 2.1. Sample

In the present study, 163 (54 athletes, 109 nonathletes) asymptomatic individuals with QRS fragmentation but without cardiac disease were consecutively recruited in the context of a heart health check. Exclusion criteria included presence of any cardiac disease and use of performance-enhancing drugs (on the basis of history taking and physical examination for the detection of signs suggestive of concealed use of performance enhancing drugs) [17]. The diagnosis of coronary artery disease in individuals older than 35 years was excluded through exercise testing or cardiac computed tomography [18].

A medical and athletic history was taken, including information about the training regimen and training age. All participants were subjected to measurement of height and body weight, resting electrocardiogram, and transthoracic echocardiography. Furthermore, 38 individuals (30 men and 8 women) were electrocardiographically assessed in standing posture and subjected to electrocardiographic exercise testing, while 13 individuals underwent cardiopulmonary exercise testing. Moreover, 44 individuals (33 men and 11 women) were reassessed with a second electrocardiogram after a follow-up period.

Written informed consent was obtained from all the participants. This study was performed in agreement with the Declaration of Helsinki and was approved by the Research Ethics Committee of University of Ioannina (13680/2024).

### 2.2. Data Collection

#### 2.2.1. Electrocardiography

A resting 12-lead electrocardiogram (Mortara RScribe) was performed in supine position with sensitivity of 10 mm/mV, paper speed of 25 mm/s, and filter range of 0.05–40 Hz. Electrocardiographic patterns were digitally evaluated by zooming in the lead of interest. The presence of narrow (<120 ms) fragmented QRS in a specific lead of the electrocardiogram was confirmed when there was a QRS with an additional R wave (R′) or notching in the nadir of the R wave or the S wave or the presence of more than one R′, with the exception of right bundle branch block pattern in V1 [2,3]. Electrocardiographic measurements included heart rate, PR interval, QRS duration, QT interval, and QRS axis. Bazett’s formula was used for calculation of corrected QT [10]. A second electrocardiogram was performed in 52 individuals (34 men and 18 women) after deep inspiration breath-hold for some seconds.

#### 2.2.2. Echocardiography

All echocardiographic images were acquired by an experienced cardiologist–ultrasonographer using a commercially available ultrasound system (Vivid I; GE Medical; Horten, Norway) with a 1.5- to 4-MHz phased-array transducer applying the same echo settings and following the same acquisition protocols. A comprehensive assessment of the structure and function of the left and right heart was undertaken in agreement with the guidelines of European Association of Cardiovascular Imaging [19,20,21]. LV end-diastolic volume (LVEDV) was estimated using the Simpson biplane method. The proximal portion of right ventricular (RV) outflow tract (RVOTprox) was measured at end-diastole in the parasternal long-axis view. Basal (RVbas), mid-cavity (RVmid), and longitudinal (RVlon) RV diameters, RV end-diastolic area (RVEDA), and RV end-systolic area (RVESA) were measured in the RV focused apical 4-chamber view. The RV fractional area change (RVFAC) was calculated as follows: [(RVEDA − RVESA)/RVEDA] × 100%. Pulmonary vascular resistance was estimated using the formula: (TRVmax/RVOTTVI) × 10 + 0.16 (in Wood units), where TRV_max_ is the peak velocity (in m/s) of the tricuspid valve regurgitant jet with continuous wave Doppler, and RVOTTVI is the time–velocity integral in the RV outflow tract [22].

Allometric scaling of echocardiographic diameters, areas, and volumes for body surface area (BSA) was performed according to the theory of geometric similarity in order to produce body size-independent variables [23,24]. The BSA was calculated using the Du Bois formula [25]. The abbreviated echocardiographic parameters indexed to BSA were distinguished by the suffix i.

#### 2.2.3. Exercise Testing

Electrocardiographic exercise testing was conducted until exhaustion on a treadmill applying the BRUCE protocol. The individuals exercised under continuous 12-lead electrocardiographic monitoring. After the end of exercise, the individual remained in a sitting position for 5 min under electrocardiographic monitoring.

Cardiopulmonary exercise testing (Ultima Series, Medgraphics, Saint Paul, MN, USA) was used for the measurement of maximum oxygen uptake (VO_2max_) during exercise and tidal volume as previously described [26].

### 2.3. Statistical Analysis

All statistical analyses were performed using the software IBM SPSS Statistics 28.0. The Kolmogorov–Smirnov test was used to verify the normality of distributions of the parameters of interest. Parameters with normal distribution were expressed as mean ± standard deviation and with nonnormal distribution as median (minimum–maximum). Comparisons of continuous variables between two independent groups were performed with the Mann–Whitney U test. The Chi-squared test was used for comparison of the presence of QRS fragmentation in a specific lead between two independent groups. We performed Wilcoxon signed-rank test to compare changes over the time in the number of leads with QRS fragmentation. We applied the McNemar test to compare changes over the time in the prevalence of QRS fragmentation in a specific lead. Analysis for exclusion of outliers using Cook’s Distance (values with Cook’s Distance > 4/n were considered outliers, where n was the number of observations) preceded correlation analysis. The univariate associations between the parameters of interest were assessed with Spearman’s correlation analysis. Multivariate logistic regression analysis was used to identify the parameters with independent associations. Receiver operating characteristic (ROC) curve analysis was performed to assess whether RVOTproxi could discriminate between athletes with QRS fragmentation in V1 and athletes without QRS fragmentation in V1. The discriminatory ability was quantified by the area under the curve (AUC). The optimal cut-off value of RVOTproxi was selected to conform with Youden’s index [J = max(sensitivity + specificity − 1)]. A two-tailed *p* value < 0.05 was considered statistically significant. Based on relevant published data, the inclusion of 74 subjects with a 1:2 enrollment ratio between the groups of athletes and nonathletes would be expected to result in 80% statistical power to detect a significant difference in the prevalence of QRS fragmentation in V1 between athletes and nonathletes [11].

## 3. Results

### 3.1. Characterization of QRS Fragmentation on Resting Electrocardiogram

Table 1 shows the demographic, training, anthropometric, and electrocardiographic characteristics of the participants. Table 2 dispays the classification of sports and sport disciplines of the studied athletes. Table 3 presents the distribution of QRS fragmentation in each specific lead among athletes and nonathletes. Among athletes, the most common lead with QRS fragmentation was III, immediately followed by V1 and aVF. The median number of leads with QRS fragmentation was 2 (1–5) in athletes. Among nonathletes, the most common lead with QRS fragmentation was III, immediately followed by aVF and aVL. The median number of leads with QRS fragmentation was 2 (1–6) in nonathletes.

Among individuals with QRS fragmentation in aVL, the distribution of QRS fragmentation in the leads of the frontal plane was as follows: III (63.3%), aVF (28.6%), I (12.2%), and II (4.1%). These percentages suggest that electrical currents corresponding to QRS fragmentation in aVL may be more commonly directed close to +150° rather than to −30°.

When we investigated whether sex influences the presence of QRS fragmentation separately in athletes and nonathletes, we found that female sex was associated with increased prevalence of QRS fragmentation in aVL among nonathletes (*p* = 0.013). However, considering that QRS fragmentation in aVL was associated with lower BMI among nonathletes (*p* = 0.026), the fact that QRS fragmentation in aVL was not associated with sex separately in the subcategories of obese and nonobese individuals suggests a possible neutral effect of sex on the prevalence of QRS fragmentation in aVL. Moreover, there was no significant association of age with the presence of QRS fragmentation in each specific lead. Furthermore, the existence of the endurance component of exercise training (i.e., mixed and endurance sports) was not associated with the presence of QRS fragmentation in each specific lead.

QRS axis correlated negatively with ΒΜΙ (rho = −0.528, *p* < 0.001), indicating a more rightward shift of electrical axis as BMI decreased. We investigated the association of BMI with the presence of QRS fragmentation in leads located on either side of III, which was the lead with the most common QRS fragmentation. The presence of QRS fragmentation in II or aVF (i.e., leads corresponding to more leftward electrical axis than III) was associated with increased BMI (26.8 ± 5.3 vs. 25.2 ± 5.1 kg/m^2^, *p* = 0.035). The presence of QRS fragmentation in aVL (i.e., lead corresponding to +150° and thus a more rightward electrical axis than III) was associated with decreased BMI (24.8 ± 4.8 vs. 26.6 ± 5.3 kg/m^2^, *p* = 0.022). The individuals with QRS fragmentation in aVL but without QRS fragmentation in aVF were characterized by lower BMI compared to the ones with QRS fragmentation in aVF but without QRS fragmentation in aVL (25.2 ± 4.8 vs. 27.3 ± 5.2 kg/m^2^, *p* = 0.028).

Among athletes and nonathletes with QRS fragmentation, the presence of QRS fragmentation in V1 was more frequent in athletes compared to nonathletes (Table 3). Among athletes, the presence of QRS fragmentation in V1 was associated with increased training age (*p* = 0.005), lower heart rate (*p* = 0.021), and more rightward QRS axis (*p* = 0.001). Among athletes, the presence of QRS fragmentation in V1 was associated with increased LVEDVi (*p* = 0.007), RVOTproxi (*p* < 0.001), RVbasi (*p* = 0.001), RVloni (*p* = 0.020), RVEDAi (*p* = 0.009), and RVFAC (*p* = 0.005). After performing multivariate logistic regression analysis in athletes, all the above-mentioned predictors of QRS fragmentation in V1, apart from RVbasi, were not significant predictors after adjustment for RVOTproxi, with the only independent predictor being the RVOTproxi. When we further performed multivariate logistic regression analysis in adult athletes, RVbas (*p* = 0.073) was not a significant predictor of QRS fragmentation in V1 after adjustment for RVOTprox, with the only independent predictor being the RVOTprox (*p* = 0.046).

**Figure 1:**
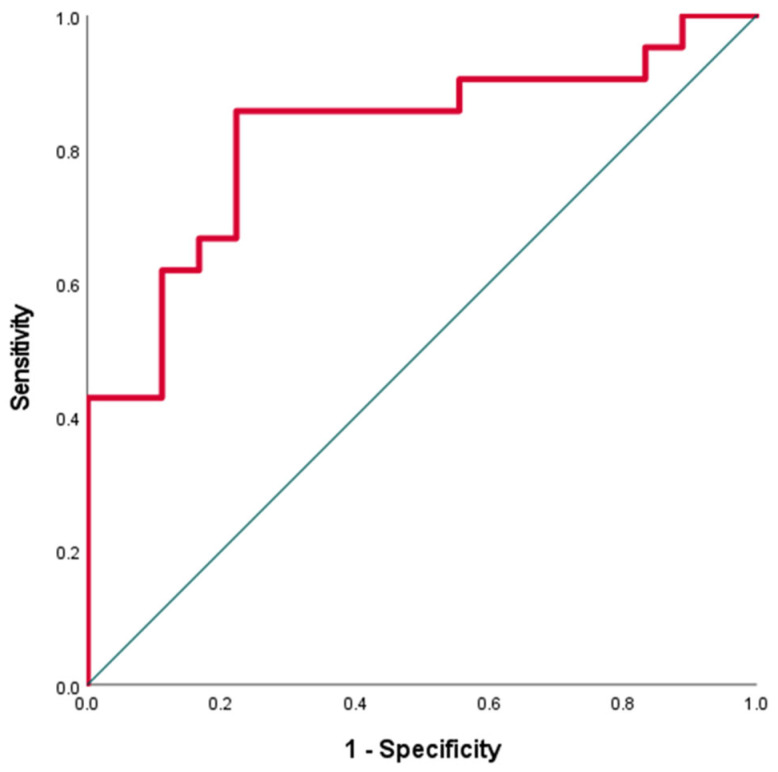
Receiver operating characteristic (ROC) curve showing the ability of RVOTproxi (right ventricular outflow tract proximal diameter indexed to body surface area) to discriminate between the athletes with QRS fragmentation in V1 and athletes without QRS fragmentation in V1. Red line: ROC curve, green line: diagonal reference line.

After applying ROC curve analysis to discriminate between the athletes with QRS fragmentation in V1 and the athletes without QRS fragmentation in V1, the AUC value was 0.820 (95%CI: 0.684–0.956) for RVOTproxi (*p* = 0.001) (Figure 1). We selected the optimal cut-off value of RVOTproxi = 0.0209 to conform with Youden’s index.

### 3.2. The Impact of Deep Inspiration on QRS Fragmentation

**Figure 2:**
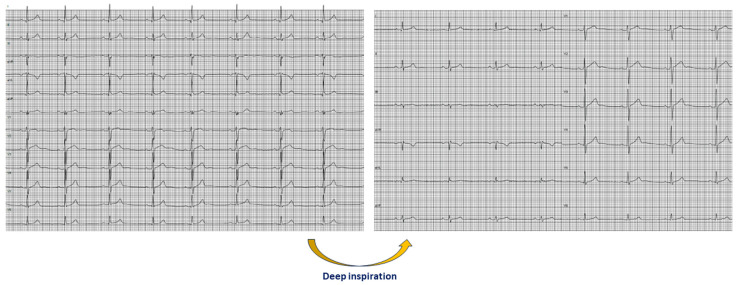
Electrocardiograms showing the disappearance of QRS fragmentation in aVF and III after deep inspiration in a 17-year-old male nonathlete.

The number of leads with QRS fragmentation decreased after deep inspiration (*p* = 0.004). The only lead in the frontal plane with a statistically significant disappearance of QRS fragmentation after deep inspiration was aVF (*p* < 0.001) (Figure 2). Considering that inspiration resulted in a rightward shift of QRS axis (*p* < 0.001), the disappearance of QRS fragmentation in aVL after deep inspiration, among individuals with baseline QRS fragmentation in aVL, was associated with greater inspiration-induced rightward shift of the QRS axis (*p* = 0.043).

**Figure 3:**
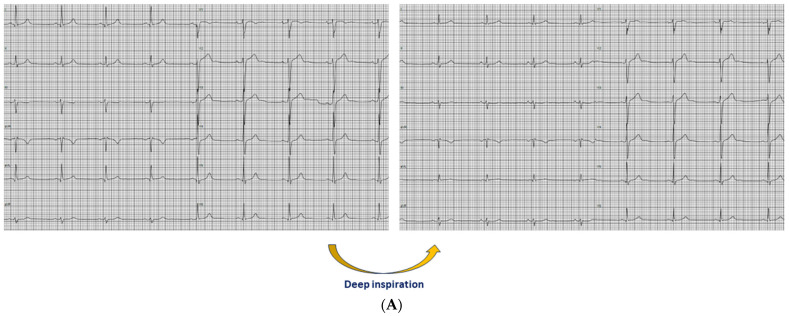

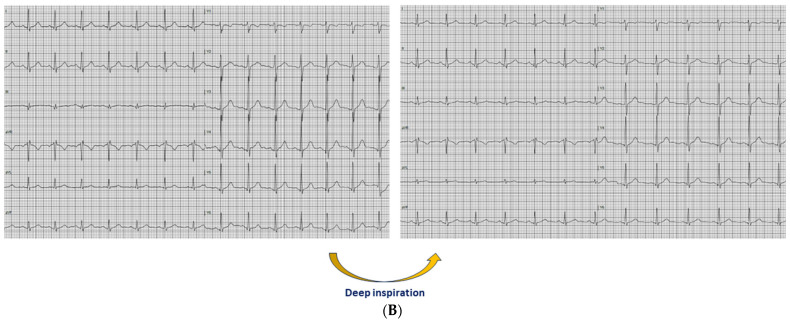
(**A**) Electrocardiograms showing the persistence of QRS fragmentation in V1 after deep inspiration in a 50-year-old male marathon runner. (**B**) Electrocardiograms showing the disappearance of QRS fragmentation in V1 after deep inspiration in a 46-year-old male nonathlete.

Among the individuals with baseline QRS fragmentation in V1, deep inspiration resulted in disappearance of QRS fragmentation more frequently in nonathletes compared to athletes (*p* = 0.003) (Figure 3). Specifically, QRS fragmentation in V1 disappeared after deep inspiration in all nonathletes but only in 20% of athletes. Among athletes, the presence of QRS fragmentation in V1 after deep inspiration was associated with increased RVOTproxi (*p* = 0.034).

### 3.3. The Influence on QRS Fragmentation by the Change in Posture from Supine to Standing

**Figure 4:**
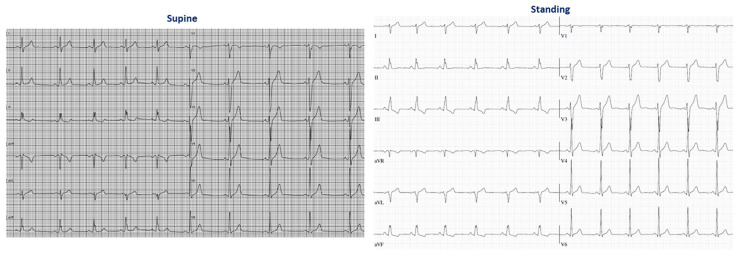
Electrocardiograms in supine and standing postures in a 40-year-old male athlete, demonstrating the new appearance of QRS fragmentation in II after standing up.

The number of leads with QRS fragmentation did not change from supine to standing posture. After performing the McNemar test for the change in the prevalence of QRS fragmentation in each lead from supine to standing posture, only the prevalence of QRS fragmentation in II increased (13.2% vs. 47.4%, *p* = 0.002) (Figure 4).

We evaluated whether the appearance of QRS fragmentation in II when standing up could be attributed to the close interrelationship between the positions of the diaphragm and heart [14,27]. The presence of QRS fragmentation in II when standing was associated with increased tidal volume (*p* = 0.016) (i.e., an index of increased vertical diameter of thorax) in adult individuals [28,29].

### 3.4. Exercise-Related Changes in QRS Fragmentation

The median number of leads with QRS fragmentation overall decreased during exercise testing from 3 (0–6) to 0 (0–4) (*p* < 0.001). Specifically, among individuals with QRS fragmentation at the baseline electrocardiogram before exercise testing, the number of leads with QRS fragmentation decreased in 90.3% of individuals, with no change in all the other individuals. The reduction in the number of leads with QRS fragmentation during exercise did not correlate with duration of exercise testing, heart rate or Mets at the time of minimum QRS fragmentation.

### 3.5. Serial Changes in QRS Fragmentation during Follow-Up

**Figure 5:**
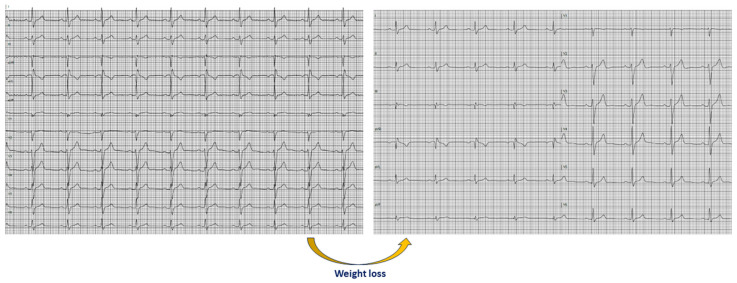
Electrocardiograms showing the disappearance of QRS fragmentation in aVF after weight loss in a 39-year-old male nonathlete. The body weight decreased from 124 kg to 114 kg, while the body mass index decreased from 34.4 kg/m^2^ to 31.6 kg/m^2^ over a period of 17 months.

After a median follow-up of 2.3 (0.8–4.9) years, all the study participants were free of cardiac events. There was a serial increase in the number of leads with QRS fragmentation in athletes (*p* = 0.018), whereas QRS fragmentation in nonathletes did not change during follow-up (*p* = 0.499). The change in the number of leads with QRS fragmentation correlated positively with training age (rho = 0.540, *p* = 0.021). The athletes in whom the number of leads with QRS fragmentation increased during follow-up were characterized by greater training age compared to the athletes without increase in the number of leads with QRS fragmentation (*p* = 0.041). Among athletes without QRS fragmentation in V1 at baseline, the new appearance of QRS fragmentation in V1 at the end of follow-up was associated with greater training age (*p* = 0.034). Among individuals with QRS fragmentation in aVF at baseline, the disappearance of QRS fragmentation in aVF at the end of follow-up was associated with greater reduction in BMI (*p* = 0.008) (Figure 5).

## 4. Discussion

They key findings of the present study include the following: (1) Among individuals with QRS fragmentation but without cardiac disease, the prevalence of QRS fragmentation in V1 was higher in athletes compared to nonathletes and was independently predicted by RVOTproxi in athletes. (2) Among individuals with QRS fragmentation in V1, deep inspiration resulted in disappearance of QRS fragmentation more frequently in nonathletes compared to athletes. (3) The presence of QRS fragmentation in the leads of the frontal plane was influenced by BMI and respiration phase.

### 4.1. Distribution of QRS Fragmentation in the Leads of Electrocardiogram

The current study found that among individuals without cardiac disease, the most common lead with QRS fragmentation was III, immediately followed by V1 and aVF in athletes and aVF in nonathletes. The generation of QRS fragmentation in the leads III and aVF in the normal heart could be plausibly explained by the helicoidal route of myocardial electrical activation of the ventricular myocardial band, since the u-turn morphology of the transition from the descending segment to ascending segment of the myocardial band corresponds to a site of sudden change in the direction of myocardial electrical activation, possibly resulting in notches on the surface electrocardiogram [30].

Taking into account that the most common lead with QRS fragmentation in the frontal plane was III, immediately followed by aVF and then by aVL, the electrical axis of QRS fragmentation was most often located between +90° and +120°. The fact that QRS fragmentation was more frequent in aVL rather than in II indicated that the electrical axis of QRS fragmentation was most often located more rightward than +105°. Hence, the electrical axis of QRS fragmentation was most commonly located between +105° and +120°.

Furthermore, we demonstrated that QRS fragmentation in V5 or V6 was extremely rare. Consistently, QRS fragmentation in lateral leads has been reported to be associated with the presence of structural heart disease and sudden cardiac death [31,32]. Thus, the detection of QRS fragmentation in V5 or V6 in a presumably healthy individual could reasonably indicate a diagnostic work up for the exclusion of cardiac disease. The benign nature of QRS fragmentation in the apparently healthy participants of the present study was further corroborated by the absence of cardiac events during the entire follow-up of several years. It should be acknowledged that an extended follow-up period is needed to better assess the prognostic significance of QRS fragmentation in individuals without known cardiac disease.

### 4.2. QRS Fragmentation in Athletes

We found that the characteristic feature of QRS fragmentation in athletes was the presence of QRS fragmentation in V1, which was associated with exercise-training-related RV dilatation, particularly of the proximal portion of RV outflow tract. These findings may reflect the fact that a dilated RV outflow tract could be associated with a greater angulation of the RV myocardial band at the transition site from the RV free wall to pulmonary conus, leading to a more sudden change in the direction of RV electrical activation in the RV outflow tract [30]. Although two previous studies have reported increased prevalence of QRS fragmentation in V1 among athletes and its association with indexes of RV dimensions, the QRS fragmentation was defined in a restrictive way as the presence of quadriphasic QRS complex without including cases with notched QRS in V1 [11,12]. Moreover, the disappearance of QRS fragmentation in V1 after deep inspiration in the healthy nonathletes of the current study could be explained by the inspiration-induced clockwise rotation of the heart minimizing the view of the RV outflow track by V1 [33]. On the other hand, the persistence of QRS fragmentation in V1 after deep inspiration in the athletes of the present study may have been caused by the sports-related RV dilatation, since the view of the dilated RV outflow tract was possibly provided by V1 even after the inspiration-induced rotation of RV outflow tract. In this respect, the identification of QRS fragmentation in V1 after deep inspiration could be considered indicative of the athlete’s heart.

We demonstrated a serial increase in the number of leads with QRS fragmentation, particularly regarding V1, in athletes during follow-up, which is possibly consistent with a process of exercise-training-related cardiac remodeling underlying QRS fragmentation. Indeed, training age was found to be closely associated with new appearance of QRS fragmentation in V1, indicating that many years of exercise training may represent a potent stimulus for induction of QRS fragmentation in athletes. To our knowledge, no previous study has investigated serial changes in QRS fragmentation during a follow-up period.

### 4.3. BMI Influence on QRS Fragmentation

The present study showed for the first time that the presence of QRS fragmentation in the leads of the frontal plane was influenced by BMI. Specifically, the higher the BMI, the more frequent the QRS fragmentation in II or aVF, implying a predominance of QRS fragmentation in more leftward-localized leads than III (III corresponds to the most common area with QRS fragmentation in the frontal plane). Conversely, the lower the BMI, the more frequent the QRS fragmentation in aVL, suggesting that electrical currents corresponding to QRS fragmentation were directed more close to +150° (i.e., more rightward than III). This great influence of QRS fragmentation in the leads of the frontal plane by BMI could be explained by the fact that increased BMI is classically associated with greater mass of abdominal adipose tissue resulting in a more leftward shift of the anatomical axis of the heart and thus more leftward shift of the myocardial electrical currents responsible for QRS fragmentation [15]. Consistently, the disappearance of QRS fragmentation in aVF during follow-up was associated with greater reduction in BMI. From this point of view, the correct interpretation of the nature of QRS fragmentation in the leads of frontal plane should take into account not only the body habitus of the individual but also the dynamic changes in body weight over time.

### 4.4. QRS Fragmentation after Deep Inspiration

In the present study, the inspiration-induced rightward shift of the QRS axis appeared to cause a decrease in the number of leads with QRS fragmentation, particularly regarding aVF. Thus, the correct evaluation of QRS fragmentation on resting electrocardiogram should be performed in a standardized manner at a specific phase of respiration. In this regard, evaluation of QRS fragmentation after deep inspiration allows a high degree of standardization and could minimize QRS fragmentation to the minimum extent possible. These considerations are particularly important in the interpretation of the serial changes in QRS fragmentation over time.

### 4.5. QRS Fragmentation upon Standing

We found that the change in posture from supine to standing caused an increase in the prevalence of QRS fragmentation in II that was associated with increased tidal volume as an index of the increased vertical diameter of the thorax [28,29]. Hence, taking into account the close interrelationship between the positions of the diaphragm and heart, the new appearance of QRS fragmentation in II when standing up could be attributed to the concomitant increase in the vertical diameter of the thorax with more vertical orientation of the heart and shift towards lead II [14,15,27].

### 4.6. Exercise-Related Changes in QRS Fragmentation

The present study found for the first time a decrease in the number of leads with QRS fragmentation during exercise testing. No previous study has investigated the changes in QRS fragmentation during exercise. We cannot completely exclude the possibility that motion-related artefacts may have hampered the detection of QRS fragmentation during exercise testing, particularly at higher intensities of exercise, contributing to the false diagnosis of disappearance of QRS fragmentation during exercise. However, considering the fact that the reduction in the number of leads with QRS fragmentation during exercise testing was not found to be associated with Mets or heart rate at the time of minimum QRS fragmentation, this explanation is less likely. An alternative mechanism may include enhancement of intraventricular conduction through sympathetic stimulation at high intensities of exercise, leading to attenuation of QRS fragmentation [16]. Further studies need to be performed to confirm these findings and elucidate the underlying mechanisms.

### 4.7. Study Strengths and Limitations

Strengths of the present study include firstly the fact that the serial changes in QRS fragmentation over a follow-up period were investigated for the first time. Moreover, we evaluated in a novel manner the dynamic alterations in QRS fragmentation following deep inspiration and standing up as well as during exercise. Thirdly, the current study assessed QRS fragmentation in each specific lead and not only in groups of contiguous leads. Furthermore, we defined the QRS fragmentation in V1 broadly, including all the subcategories of QRS fragmentation, as opposed to the restrictive definition of previous studies investigating only the presence of quadriphasic QRS complex without including cases with notched QRS in V1 [11,12]. Finally, the present study investigated QRS fragmentation in individuals with a wide age range, including not only children and adolescents but also elderly individuals. This characteristic has profound impact on the generalizability of the results. Similarly, we evaluated individuals of both sexes and athletes of various sport disciplines, further contributing to the generalizability of the results.

The results of our study should be interpreted in light of some limitations. Firstly, the number of athletes was lower than the number of nonathletes resulting in decreased power of the study to detect significant differences between athletes and nonathletes. Secondly, cardiac magnetic resonance imaging was not performed in the present study and thus the presence of myocardial scars cannot be totally ruled out. Nevertheless, taking into account that no red flags or suspicious findings were noticed on electrocardiogram, echocardiogram, and exercise testing in any participant, myocardial scars were reasonably considered very unlikely. Thirdly, the current study did not evaluate the potential confounding effect of medication use, diet, and stress on the presence of QRS fragmentation.

## 5. Conclusions

In conclusion, the characteristic feature of QRS fragmentation in athletes was the presence of QRS fragmentation in V1, which was associated with RVOTproxi. Τhe persistence of QRS fragmentation in V1 after deep inspiration could serve as a relatively specific marker of exercise-training-related cardiac adaptation. The presence of QRS fragmentation in the leads of the frontal plane may be influenced by BMI and respiration phase. Further studies need to be performed to investigate the extent of the reversibility of QRS fragmentation in athletes after a period of detraining.

## Figures and Tables

**Table 1:** The demographic, training, anthropometric, and electrocardiographic characteristics of the participants.

	Athletes (*n* = 54)	Nonathletes (*n* = 109)
Sex (men/women)	45/9	69/40
Age (years)	28 ± 17	52 ± 19
Classification of sports (skill/power/mixed/endurance)	0/12/25/17	-
Training age (years)	12 ± 11	-
Training volume (h/week)	6.4 ± 3.4	-
Body mass index (kg/m^2^)	23.4 ± 5.0	27.3 ± 4.9
Heart rate (bpm)	67 ± 15	69 ± 12
PR (ms)	157 ± 28	161 ± 26
QTc (ms)	400 ± 21	410 ± 21
QRS duration (ms)	102 ± 14	98 ± 12
QRS axis (°)	44 ± 39	20 ± 31

**Table 2:** Classification of sports and sport disciplines.

Classification of Sports	Sport Disciplines	Athletes
Skill	-	0
Power	Weight lifting	6
	Ballet/rhythmic gymnastics	3
	Short-distance running	1
	Long jump	1
	Surfing	1
Mixed	Soccer	18
	Basketball	3
	Volleyball	3
	Tennis	1
Endurance	Long/middle-distance running	15
	Cycling	1
	Rowing	1

**Table 3:** The distribution of QRS fragmentation in each specific lead among athletes and nonathletes.

	Athletes (*n* = 54)	Nonathletes (*n* = 109)	*p* Value
I	1 (1.9%)	6 (5.5%)	0.279
II	4 (7.4%)	20 (18.3%)	0.064
III	34 (63.0%)	67 (61.5%)	0.853
aVR	0 (0%)	0 (0%)	1.000
aVL	18 (33.3%)	31 (28.4%)	0.521
aVF	23 (42.6%)	60 (55.0%)	0.134
V1	27 (50.0%)	20 (18.3%)	<0.001
V2	4 (7.4%)	6 (5.5%)	0.634
V3	3 (5.6%)	2 (1.8%)	0.195
V4	1 (1.9%)	1 (0.9%)	0.610
V5	0 (0%)	1 (0.9%)	0.480
V6	0 (0%)	1 (0.9%)	0.480

Chi-squared test was used for the comparison of the presence of QRS fragmentation in a specific lead between athletes and nonathletes. *p* values in bold indicate statistical significance.

## Data Availability

Dataset available on request from the authors.
